# An integrated proteomic and transcriptomic signature of the failing right ventricle in monocrotaline induced pulmonary arterial hypertension in male rats

**DOI:** 10.3389/fphys.2022.966454

**Published:** 2022-11-01

**Authors:** Charles Colin Thomas Hindmarch, Lian Tian, Ping Yu Xiong, Francois Potus, Rachel Emily Teresa Bentley, Ruaa Al-Qazazi, Kurt W. Prins, Stephen L. Archer

**Affiliations:** ^1^ QCPU, Queen’s Cardiopulmonary Unit, Translational Institute of Medicine (TIME), Department of Medicine, Queen’s University, Kingston, ON, Canada; ^2^ Department of Medicine, Queen’s University, Kingston, ON, Canada; ^3^ Pulmonary Hypertension Research Group, Centre de Recherche de l’Institut Universitaire de Cardiologie et Pneumologie de Quebec, Quebec City, QC, Canada; ^4^ Cardiovascular Division, Lillehei Heart Institute, Department of Medicine, University of Minnesota, Minneapolis, MN, United States

**Keywords:** mitochondria, SU5416-chronic hypoxia PAH model, tenascin-C (TNC), periostin (POSTN), thrombospondin-4 (Thbs4), heme oxygenase 1 (HMOX1), glutathione S-transferase zeta 1 (Gstz1), S100 calcium-binding protein A4 (S100A4)

## Abstract

**Aim:** Pulmonary arterial hypertension (PAH) is an obstructive pulmonary vasculopathy that results in death from right ventricular failure (RVF). There is limited understanding of the molecular mechanisms of RVF in PAH.

**Methods:** In a PAH-RVF model induced by injection of adult male rats with monocrotaline (MCT; 60 mg/kg), we performed mass spectrometry to identify proteins that change in the RV as a consequence of PAH induced RVF. Bioinformatic analysis was used to integrate our previously published RNA sequencing data from an independent cohort of PAH rats.

**Results:** We identified 1,277 differentially regulated proteins in the RV of MCT rats compared to controls. Integration of MCT RV transcriptome and proteome data sets identified 410 targets that are concordantly regulated at the mRNA and protein levels. Functional analysis of these data revealed enriched functions, including mitochondrial metabolism, cellular respiration, and purine metabolism. We also prioritized 15 highly enriched protein:transcript pairs and confirmed their biological plausibility as contributors to RVF. We demonstrated an overlap of these differentially expressed pairs with data published by independent investigators using multiple PAH models, including the male SU5416-hypoxia model and several male rat strains.

**Conclusion:** Multiomic integration provides a novel view of the molecular phenotype of RVF in PAH which includes dysregulation of pathways involving purine metabolism, mitochondrial function, inflammation, and fibrosis.

## 1 Introduction

Pulmonary hypertension (PH) is defined as a mean pulmonary artery pressure (mPAP) > 20 mmHg. The World Health Organization broadly organizes PH into five groups, based on clinical characteristics, histology and etiology (Ryan et al., 2012). Group 1 PH, or pulmonary arterial hypertension (PAH) is a syndrome diagnosed by the presence of PH and elevated pulmonary vascular resistance (PVR >3 Wood Units) in the absence of left heart disease, lung disease, hypoxia and venous thromboembolism. While there are inherited forms of PAH, the majority of cases are either idiopathic or associated with connective tissue diseases, like scleroderma.

In Group 1 PH, the primary pathology is located in the arterial portion of the pulmonary circulation where excessive pulmonary vasoconstriction and adverse obstructive remodelling, largely the latter, increases right ventricle (RV) afterload, leading to right ventricular failure (RVF) (Fletcher et al., 2013). Current therapies for PAH target the pulmonary vasculature, not the RV. While these therapies improve functional capacity, mortality rates remain high primarily due to RVF (Vonk Noordegraaf et al., 2011). Although RV function is the major determinant of survival in PAH, as it is in other PH groups (Thenappan et al., 2010), RVF remains understudied in both preclinical models and PAH patients. RV afterload increase in PAH is initially countered with an adaptive hypertrophic response (RVH). We have previously compared models of RV pressure overload in rats that develop nearly identical RVH and RV mass but are either adaptive, meaning relatively resistant to RVF (e.g., pulmonary artery banding models, PAB) or maladaptive, prone to RVF (e.g., MCT-PAH). The PAB rats do not proceed to RVF over a period of several months whilst the MCT rats, despite similar RV pressures and RVH, develop lethal RVF in ∼1 month (Piao et al., 2012). As in patients, the reasons for variable adaptation despite similar RV pressure and RVH remain elusive.

Technetium sestamibi nuclear scintigraphy and 18fluorodeoxyglucose positron emission tomography in patients with PAH has shown that the hypertrophied RV is ischemic and manifests a metabolic switch toward a more glycolytic metabolic state, with suppression of glucose oxidation (the Warburg phenomenon) (Gómez et al., 2001; Oikawa et al., 2005) This maladaptive mitochondrial metabolic phenotype is also observed in MCT-PAH RVs (Piao et al., 2010a; Piao et al., 2010b; Fang et al., 2012). Although individual targets that explain decompensation have been described in the literature, such as inhibition of glucose oxidation, abnormal fatty acid oxidation and induction of Glutaminolysis (Piao et al., 2010a; Piao et al., 2010b; Fang et al., 2012), we decided to apply a layered multi-omic approach to phenotyping the decompensated RV in MCT-PAH because of its ability to provide a broad unbiased profile of the RV’s response to PAH.

Recent RNAseq studies from our lab revealed changes in gene expression associated with RVF in PAH. RNAseq is a method by which RNA from biological samples can be prepared into fragmented libraries so that the sequence of each fragment can be read (Johnson et al., 2015). These reads are aligned back to the reference genome in order for transcript assembly and counting to be achieved so that differentially regulated genes can be resolved. In the MCT RV, we identified 2,546 transcripts that were significantly and differentially expressed (1,457 upregulated and 1,089 downregulated). In the MCT RV, the most significantly enriched terms converged on functions or gene ontology (GO) pathways, including mitochondria/metabolic, fibrosis and inflammation (Potus et al., 2018).

We now define the proteomic signature of the decompensated male PAH RV within a separate, independent cohort of male rats with MCT PAH, and integrate changes in protein and expression to better understand the response of the RV in PAH. Because we already have a catalogue of transcriptomic data from the RV of an independent cohort of MCT and control animals, we performed data integration that not only validates both datasets but provides us with a comprehensive signature of decompensated RVF at the mRNA and protein level. Finally, we compared the most highly enriched mRNA and protein targets from our cohort to published microarray data from male SU5416-chronic hypoxia PAH model (Suen et al., 2019), performed in a different rat strain by an independent research group. Our goal was to determine, and independently validate whether the top dysregulated mRNA/protein pairs in our MCT RVs were similarly regulated in other models of severe PAH-induced RVF in male rats.

## 2 Materials and methods

### 2.1 Animals

All experimental protocols were performed under the approval of the Queen’s University Animal Care Committee and the University Research Ethics Board (2017–1714). Male Sprague Dawley rats (Charles River) were used in this experiment. The tissue used in this experiment was extracted from sham control animals (*n* = 5) and sham MCT animals (*n* = 5). These animals have been repurposed from a discrete experiment that involved supra-coronary aortic banding (SAB) experiment (Tian et al., 2020a); these shams were therefore subject to surgery and recovery but were not banded. We account for the use of these sham animals and demonstrate that the expression of critical genes is comparable to unoperated control animals.

Briefly, following anesthetic, (2–2.5% isoflurane), rats were mechanically ventilated (60–70 breath/minute, tidal volume of 6.0 ml/kg) and the intercoastal muscle was dissected to allow for the visualization of the ascending aorta and then the incision was closed without intervention. Standard postoperative care was provided to animals (buprenorphine 0.05 mg/kg). Following recovery from sham surgery, rats received a single subcutaneous injection of either phosphate-buffered saline (PBS; 2 ml/kg, *n* = 5) or MCT (60 mg/kg, *n* = 5). Following sacrifice, the RV was isolated, dissected and snap frozen. qPCR validation was performed on this sham MCT tissue (*n* = 3), and from non-sham control (*n* = 5) and non-sham MCT animals (*n* = 5).

### 2.2 Echocardiography

At week four post injection, echocardiography was performed in animals anesthetized with isoflurane (2–2.5%) using a high-frequency ultrasound system (Vevo 2,100; Visual Sonics, Toronto, ON, Canada), as described (Tian et al., 2020a). Briefly, pulsed-wave Doppler was obtained to measure pulmonary artery acceleration time (PAAT) and systolic velocity time integral (VTI) in the pulmonary outflow tract. The main pulmonary artery (PA) inner diameter (ID) during mid-systole was obtained from an M-mode image of the pulmonary artery. The M-mode image of long-axis view of RV free wall (RVFW) was obtained to measure the diastolic and systolic thickness of RVFW. RVFW systolic thickening was calculated as (RVFW_systole_-RVFW_diastole_)/RVFW_diastole_. Tricuspid annular plane systolic excursion (TAPSE) was measured from the apical four-chamber view.

### 2.3 Catheterization

Cardiac catheterization was performed as described (Tian et al., 2020a). Briefly, left heart catheterization (*via* right carotid artery) and right heart catheterization (*via* right jugular vein) (LHC and RHC respectively) were performed in closed-chest animals that were anesthetized with isoflurane (2.5–3.0%). We used micromanometer, a high-fidelity 1.9-F rat pressure-volume catheter (Transonic, London, ON, Canada). Stroke volume (SV), cardiac output (CO), and heart rate (HR) were obtained from LHC. RV systolic pressure (RVSP) and RV end-diastolic pressure were obtained from RHC. Pulmonary vascular resistance (PVR) was calculated as (mPAP-LVEDP)/CO, where LVEDP is left ventricular end-diastolic pressure. Mean pulmonary artery pressure (mPAP) is estimated as 0.6 * RVSP+2 (Chemla et al., 2004).

### 2.4 Protein extraction and mass-spectrometry

Following harvest, RV was flash-frozen and stored at −80°C until required. Protein digestion and mass spectrometry analyses were performed by the Proteomics Platform of the CHU de Quebec Research Center (Quebec, Canada). Briefly, 20ug proteins were resuspended in ammonium bicarbonate ((50 mM)/deoxycholate (1%)) and disulfide bridges were reduced by dithiothreitol and alkylated with iodoacetamide. Proteins digested with Trypsin (1:50) were purified using StageTips C18 and dried using a speed vac. Samples were then resuspended in liquid chromatography (LC) loading solvent and 1 µg was injected and analyzed by Liquid Chromatography with Tandem Mass Spectrometry (LC-MSMS) using an Orbitrap Fusion Tribrid system (Thermo, Waltham, MA). LC runs (150 min gradients) were used and the MS was operated in a data dependent acquisition (DDA) mode. Identification and label free quantification were performed using MaxQuant software v1.6.10.43 using the *Rattus norvegicus* database and data was reported using UniProt ID’s.

### 2.5 Ribonucleic acid extraction and quantitative polymerase chain reaction validation

RNA was extracted from flash frozen rat RV tissue stored at −80°C . There were five controls (PBS-treated), five MCT-treated, and three Sham-MCT. Tissue was ground with mortar and pestle with liquid nitrogen. Samples were lysed in 1,000 μL TRI Reagent^®^ (Sigma-Aldrich, Oakville, Ontario, CA). RNA was isolated using Zymo Direct-zol RNA MiniPrep (Zymo Research, California, United States), according to manufacturer’s protocols, and quantified using the DropSense 16 (Trinean, Pleasanton, California United States) according to manufacturer’s protocols. Complementary DNA (cDNA) was synthesized using qScript™ cDNA Supermix (Quantabio, Beverly, Massachusetts, United States) according to manufacturer’s protocol, with 500 ng RNA input per sample. cDNA was then quantified using the DropSense 16, and all samples were adjusted to the lowest concentration.

qPCR was conducted using TaqMan™ probes (ThermoFisher Scientific, Mississauga, Ontario, CA) and PerfeCTa Fastmix II (Quantabio, Beverly, Massachusetts, United States) according to manufacturer’s protocol, using the QuantStudio™ 3–96-well 0.2 ml Block (ThermoFisher Scientific, Mississauga, ON, CA). All probes for target genes used FAM as the reporter and NFQ-MGB as the quencher. Each well was multiplexed with a probeset for Eukaryotic 18S rRNA Endogenous Control (ThermoFisher Scientific, Mississauga, ON, CA) with the reporter VIC and quencher TAMRA. In order to validate gene expression in the sham MCT animals, we selected the following genes for validation in both shams and control MCT compared with control: Postn (Rn01494627_m1), Ltbp2 (Rn00572063_m1), and Nppa (Rn00664637_g1). Data was analyzed using the 2∆∆Ct method (Pfaffl, 2001), and statistics were performed using Prism (GraphPad Software, LLC). Normality was confirmed with Shapiro-Wilk test and we analyzed Postn using a one-way ANOVA with Tukey multiple comparisons test and analyzed Nppa and Ltbp2 (both had unequal standard deviations) using Brown-Forsythe and Welch ANOVA with Dunnett’s T3 multiple comparisons test.

### 2.6 Data analysis and transcriptome integration

Data was preprocessed and handled within the R environment (Vienna, Austria). Briefly, zero sum numbers were replaced by 0.01 percentile value and only proteins satisfying at least three positive values out of five progressed to analysis. Mean intensity per group, intensity ratio (and log2 ratios), and z-score were calculated and a linear model for microarray data (Limma; Ritchie et al., 2015) was used to calculate *p*-value changes. Benjamini–Hochberg corrected q-values were calculated for each protein expression between control and MCT groups. BioMart (Durinck et al., 2009) in R was used to convert the UniProt IDs to Ensembl IDs and this list was hand-curated to ensure that UniProt ID’s that were not converted were included for transcriptome integration of genes and proteins based on common annotation. Finally, this list was merged with our published (Potus et al., 2018) list of differentially regulated genes in the MCT RV compared to controls in order to identify common transcripts and proteins. Functional networks using Gene Ontology were performed using the R package, Cluster Profiler (Yu et al., 2012), Venn diagrams were generated with online software (Heberle et al., 2015).

## 3 Results

### 3.1 Animal physiology

We made use of tissue extracted from the RV of male Sprague Dawley rats that had received sham surgery and then exposed to either vehicle (phosphate-buffered saline; PBS) or MCT. This data is published elsewhere (Tian et al., 2020a). Because we used these sham operated animals to reduce unnecessary sacrifice of animals, we demonstrate that gene expression in the sham-MCT group was consistent with the non-surgical MCT animals. To do this, we performed qPCR to interrogate the regulation of three transcripts, chosen because they were identified as robustly regulated at the transcript level in the MCT RV in our previously published work (Tian et al., 2020b), (Postn, Nppa & Ltbp2; Supplementary Figure S1). Relative to control RVs, there were no significant differences in the expression of these genes between the naïve MCT rats, and those exposed to sham surgery.

In addition, our hemodynamic data demonstrates pulmonary vascular disease in the MCT-sham group compared to their controls (PBS-Sham). This is presented as an increase in mPAP, RVSP and PVR (*p* = 0.005, *p* = 0.005, *p* = 0.014) respectively and a decrease in PAAT (*p* < 0.0001). There is also RV hypertrophy (RVH) in the MCT group shown as a reduction in RVFWT, and an increase in Fulton index when compared to controls (*p* < 0.0001, *p* = 0.0001) respectively. RVH was also associated with a significant reduction in RV function marked by reduced TAPSE (*p* < 0.0001) and CO (*p* = 0.0062) and increased RVEDP (*p* = 0.017) when compared to control. Ventricular to arterial (RV-PA) coupling was also assessed by measuring the ratio of TAPSE/RVSP (Tello et al., 2019). Our data shows that RV-PA coupling is significantly decreased in MCT (*p* < 0.0001) group which further confirms RV failure (RVF) in MCT-sham rats compared to PBS-Sham. Cardiac ultrasound and RHC data are all presented in (Table 1). Regarding the LV function, our PV-loop data shows a significant reduction in left ventricular systolic pressure (LVSP) and a non-significant decrease in left ventricular end-diastolic pressure (LVEDP) in MCT rats when compared to controls (*p* = 0.027, *p* = 0.145 respectively; Supplementary Figure S3). We did not measure LV function using cardiac ultrasound.

**TABLE 1:** Haemodynamic assessment of sham Sprague Dawley rats injected with either PBS (2 ml/kg; *n* = 5) or monocrotaline (60 mg/kg; *n* = 5) in order to induce pulmonary arterial hypertension (PAH).

Rat cardiac function information			
	Sham	MCT	*p*-value
**Cardiac Ultrasound**			
^a^RVFWT%	121.6 ± 3.868	23.8 ± 2.939	<0.0001****
^b^TAPSE (mm)	3.262 ± 0.06	2.040 ± 0.04	<0.0001****
^c^PAAT (ms)	34.0 ± 1.225	19.8 ± 2.950	<0.0001****
RHC			
^d^RVSP (mmHg)	23.30 ± 2.020	56.99 ± 7.265	0.005**
^e^mPAP (mmHg)	16.21 ± 1.231	36.77 ± 4.432	0.005**
^f^RVEDP (mmHg)	1.505 ± 0.458	5.954 ± 1.224	0.017*
^g^PVR (mmHg/ml/min)	0.046 ± 0.022	1.462 ± 0.310	0.014*
^h^CO (ml/min)	107.5 ± 13.1	49.91 ± 8.36	0.0062**
^i^SV (ul)	301.8 ± 39.37	96.08 ± 17.77	0.001**
HR (beats/min)	335.4 ± 13.69	268.0 ± 13.74	0.008**
TAPSE/RVSP	0.142 ± 0.01	0.040 ± 0.004	<0.0001****
Anatomical parameter			
Fulton Index	0.287 ± 0.01	0.712 ± 0.06	0.0001***

^a^ RVFWT% = Right ventricular free wall thickness change.

^b^ TAPSE = Tricuspid Annular Plane Systolic Excursion,

^c^ PAAT = pulmonary artery acceleration time.

^d^ RVSP = Right Ventricular Systolic Pressure.

^e^ mPAP = mean Pulmonary Arterial Pressure.

^f^ RVEDP = Right ventricular End Diastolic Pressure.

^g^ PVR = Pulmonary Vascular Resistance.

^h^ CO = Cardiac Output.

^i^ SV = Stroke Volume, HR = Heart rate. Values are presented as Mean ± SEM. Parametric unpaired (t test) was used to compare Sham and MCT rats: *(p ≤ 0.05), **(p ≤ 0.01), ***(p ≤ 0.001), ****(p < 0.0001).

### 3.2 Mass spectrometry

**FIGURE 1:**
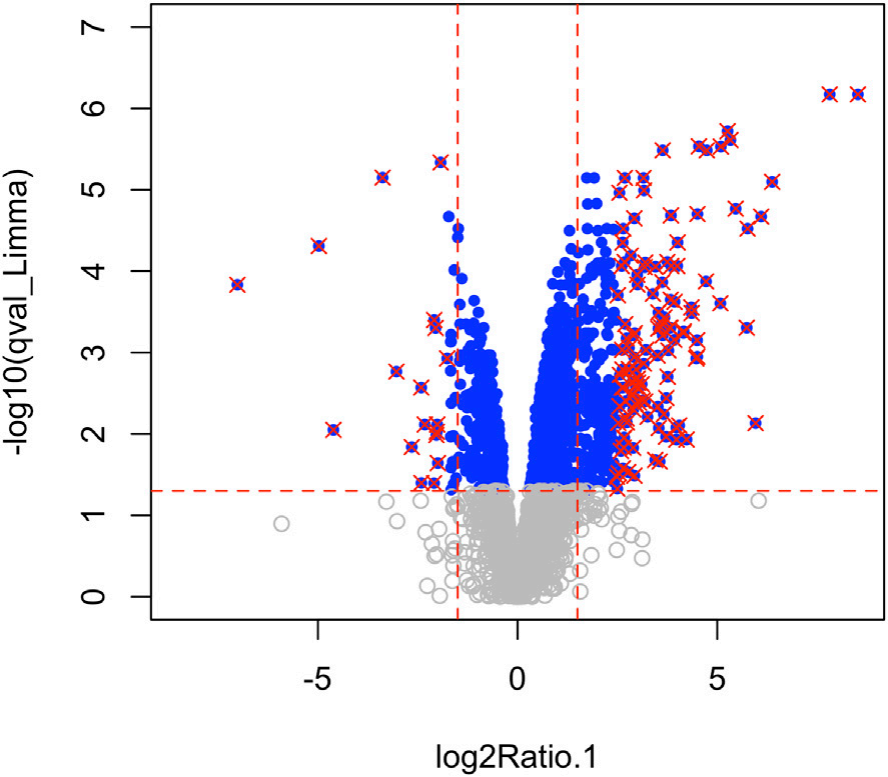
Mass spectrometry revealed 3,134 proteins, of which 1,277 were considered differentially regulated (q-value<0.05; blue points) and of these, 137 proteins satisfied z-score criteria (±1.96; red X).

**FIGURE 2:**
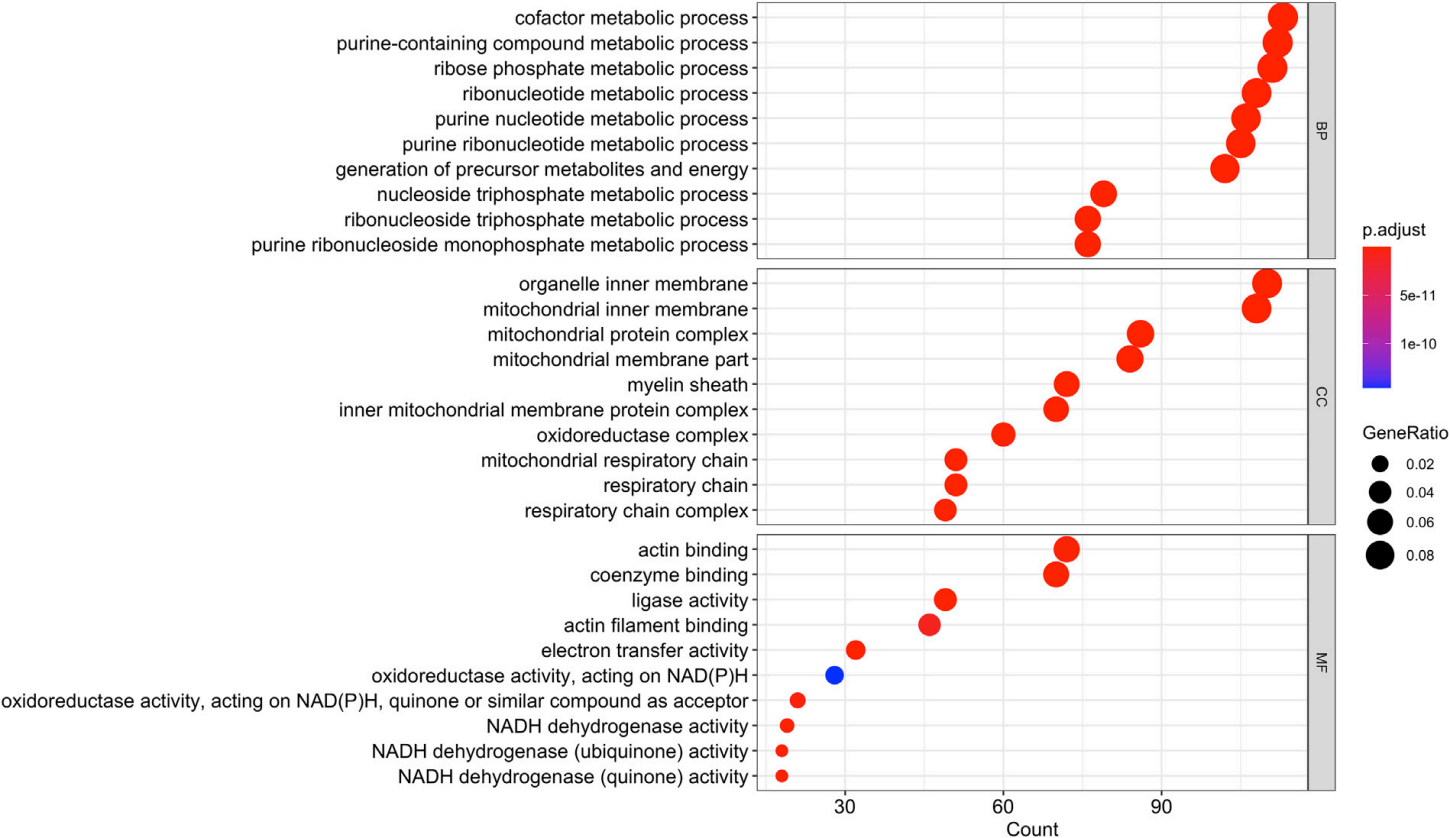
Gene Ontology analysis of the 1,277 differentially regulated proteins (q-value<0.05) between the monocrotaline right ventricle (RV) and sham RV revealed an enrichment in biological processes involved in purine and ribose metabolism, cellular component terms involving mitochondrial and respiratory chain, and molecular functions involving cellular respiration.

Using Mass Spectrometry, 1,277 proteins were found to be significantly (corrected *p*-value<0.05) differentially expressed in MCT *versus* control RV (Figure 1; Supplementary Data S1). When we applied a more stringent z-score cut-off (±1.96 σ) to this list of proteins, we identified 137 significantly dysregulated proteins. We evaluated the functional roles that these proteins played in the RV using pathway analysis. Using the list of 1,277 proteins, we performed a Gene Ontology (GO) analysis using Cluster Profiler (Yu et al., 2012) and revealed functional groups of proteins that were significantly enriched, as assessed using a Benjamini–Hochberg multiple test correction (*p* ≤ 0.05). We further classified groups of regulated proteins according to their cellular component (CC; 175 enriched terms), biological process (BP; 439 enriched terms) and imputed molecular function (MF; 125 enriched terms). (Figure 2; Supplementary Data S2).

### 3.3 Proteome: Transcriptome integration

**FIGURE 3:**
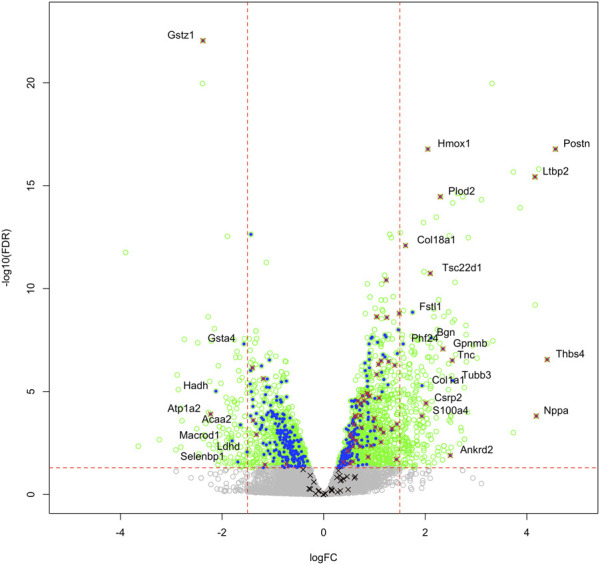
Transcriptome and proteome integration: We compared our list of 1,277 significant proteins to our previously published list of 2,543 significantly regulated genes (green open circles), identified in the RVs of an independent cohort of MCT *versus* Control rats. We identified an overlap of 410 proteins that are also regulated at the mRNA level (blue filled circles), many of which also satisfy the z-score cutoff in our proteomic data (red x). Some targets were regulated at the protein level, but not mRNA level (black x). Labelled gene/proteins satisfy a 1.5-fold change cutoff in mRNA and protein expression, respectively.

**FIGURE 4:**
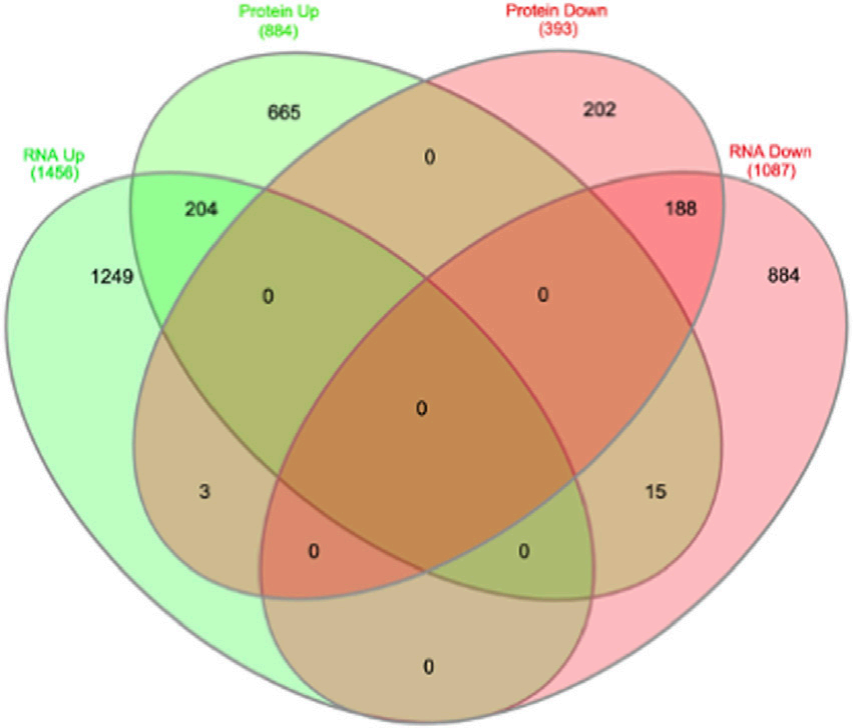
Overlap of transcriptome and proteome data overlap identifies 410 common targets, of which 18 are contra-regulated.

**FIGURE 5:**
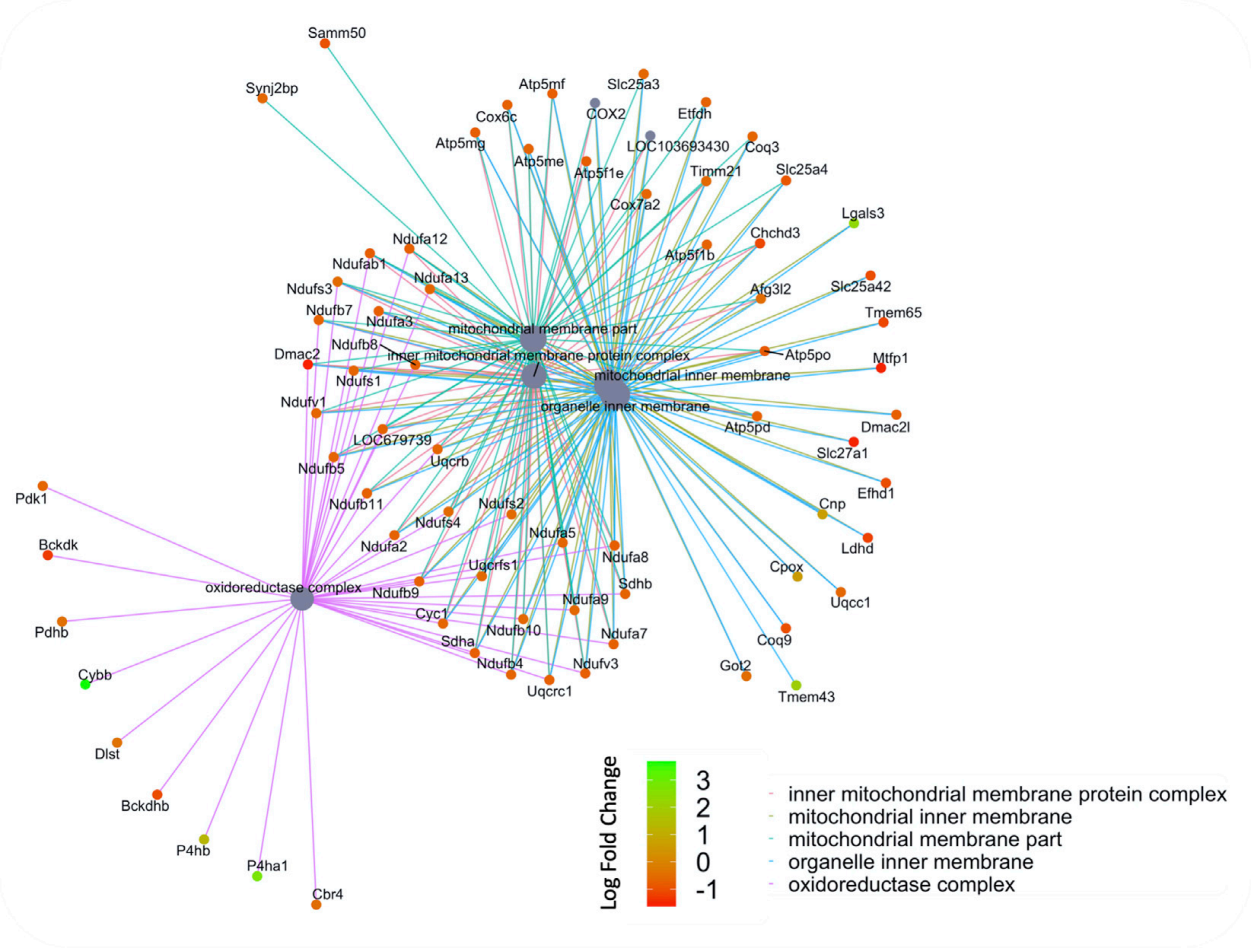
Network of GO enriched terms within the Cellular Component Domain. An enrichment of terms describing “mitochondria” was resolved when we performed functional analysis on the 410 proteins that are also differentially regulated at the transcript level.

**FIGURE 6:**
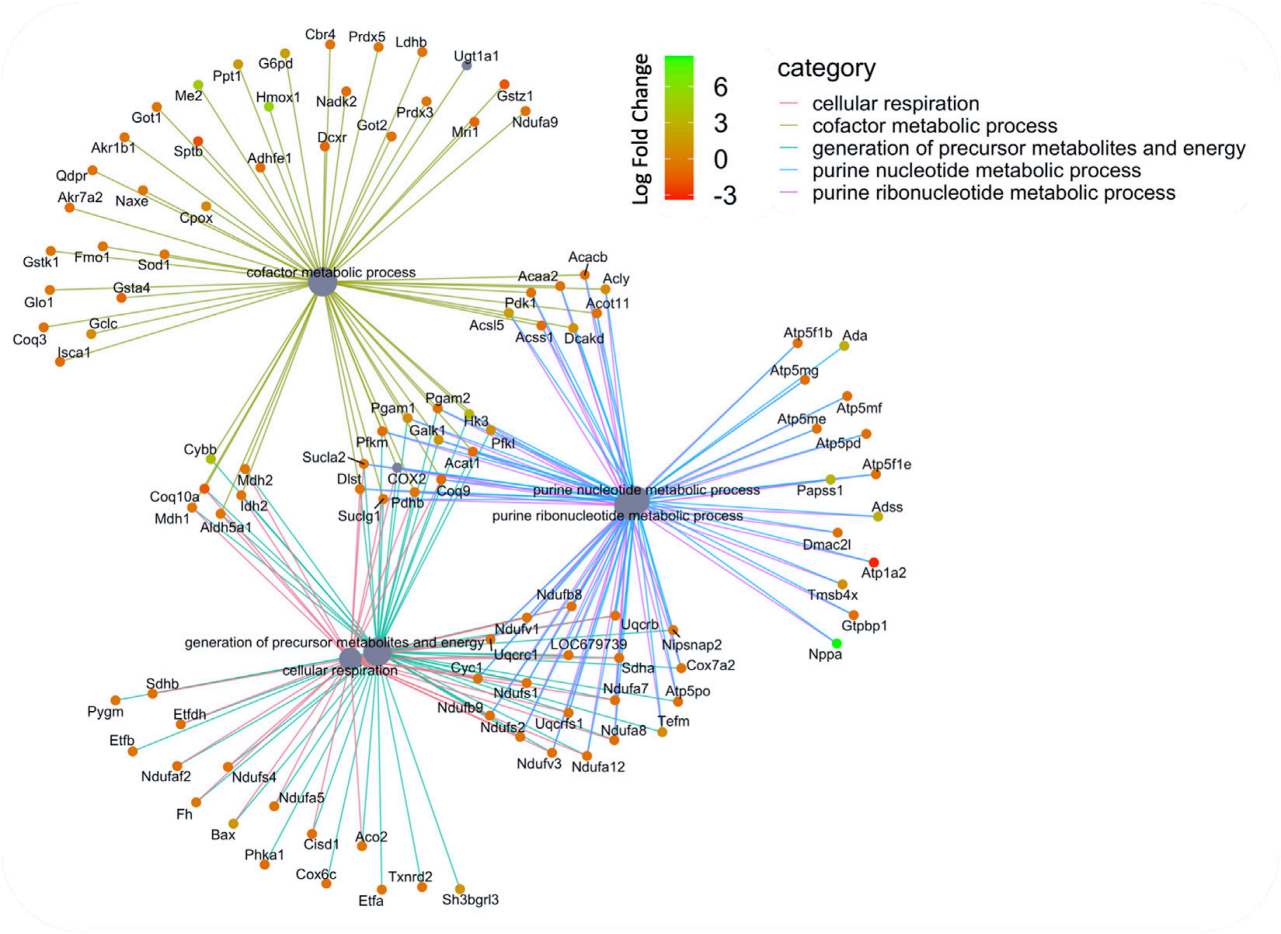
Network of GO enriched terms within the Molecular Function Domain. An enrichment of terms describing ‘cellular respiration’ and “purine metabolism” was resolved when we performed functional analysis on the 410 proteins that are also differentially regulated at the transcript level.

**FIGURE 7:**
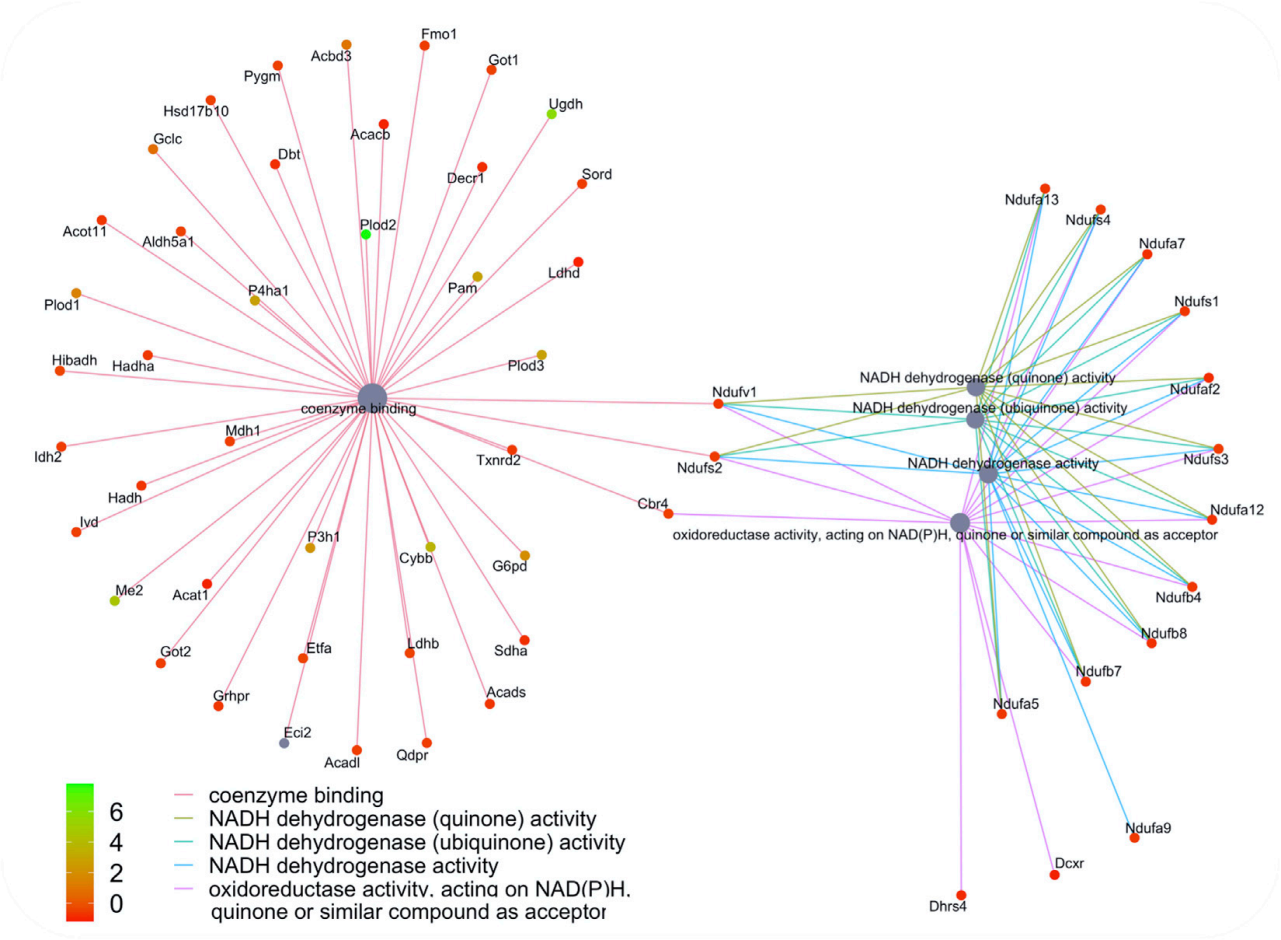
Network of GO enriched terms within the Biological Process Domain. An enrichment of terms describing “NADP dehydrogenase” was resolved when we performed functional analysis on the 410 proteins that are also differentially regulated at the transcript level.

We then compared these GO protein lists to our previously published transcriptome wide data obtained from the RV of a separate cohort of rats with MCT PAH and RVF, as described (Potus et al., 2018). This revealed that of the 1,277 proteins differentially regulated in the current MCT RV cohort, 410 transcripts from a prior MCT cohort were commonly regulated, meaning both the protein and transcript were concordantly increased or concordantly decreased in RVF (Figures 3, 4; Supplementary Data S1). When we compared the regulation direction of these protein: transcript pairs, all but 18 (Figure 4; Supplementary Table S3) of the pairs were concordant (i.e., regulated in the same direction). When we compared these 410 targets, identified by being concordantly regulated at the transcript and protein levels, to RNAseq data published by (Park et al., 2021), which they derived using the MCT PAH model, we found tightly overlapping expression patterns (97% overlap between lists) (Supplementary Figure S2). When our list of 410 similarly regulated protein: transcript pairs was analyzed using functional annotation, there was an enrichment of terms within the three GO domains; Cellular Component (Figure 5; Supplementary Data S3), Biological Process (Figure 6; Supplementary Data S3) and Molecular Function (Figure 7; Supplementary Data S3), with a focus on pathways involving purine metabolism, mitochondrial function and cellular respiration.

### 3.4 Remining published public data

Lastly, we prioritized protein targets that satisfied the most stringent statistical analysis (qval-Limma *p* < 0.05, z-score ± 1.96) which were also highly enriched transcripts in our previously published RNAseq data that was performed on an independent cohort of animals (Potus et al., 2018). This revealed 15 targets that are commonly regulated protein: transcript pairs (Table 2). This list was compared to recently published microarray data from Suen et al. which relied upon male Fischer and male Sprague Dawley (SD) rats exposed to the VEGF2 receptor antagonist Sugen5416 + hypoxia (Suen et al., 2019) which prompts severe PAH in the Fisher (elevated BNP, RVSP, RVFWT, fulton index), but less severe PAH in the SD rats. In total, there were nine commonly regulated genes in the male Fischer RV and six commonly regulated genes in the male SD RV. We also compared this list of 15 protein: transcript pairs to data published by Park et al., which looked at the RV in response to either MCT or Sugen5416 + hypoxia (Park et al., 2021) (both resulting in elevated RVF). In total, 14 of our 15 protein: transcript targets were also robustly regulated in the Park et al. MCT male SD rats and nine commonly regulated targets in the male Sugen5416 + hypoxia SD RV. As previously mentioned, there is a 97% overlap between the Park male MCT RV transcriptome data and our list of 410 concordantly regulated protein: transcript pairs in the male RV.

**TABLE 2:** List of highly enriched proteins (q-value <0.05 & z-score ± 1.96) that also have highly enriched transcripts (FDR <0.05 & fold change >1.5) in our previously published work (Potus et al., 2018), in data published from the right ventricle (RV) from Fischer rats + Sugen5416 + hypoxia (SUHx) or Sprague Dawley (SD) rats + SUHx (Suen et al., 2019), or from RV SD + MCT rats and SD + SUHx (Park et al., 2021).

Annotation		Protein			Potus et al. RNA		Suen et al. RNAseq		Park et al. RNAseq	
Description	Symbol	zscore	Log FC	q-value limma	MCT Log FC	MCT p-value	Fischer + SUHx FC	SUHx FC	SD + SUHx FC	SUHx FC
natriuretic peptide A	Nppa	7.55	8.52	6.73E-07	4.19	1.55E-04	47.56	-	—	—
thrombospondin 4	Thbs4	5.56	6.38	8.00E-06	4.4	2.80E-07	16.51	10.11	1.8	1.3
periostin	Postn	3.83	4.5	1.99E-05	4.56	1.67E-17	8.84	7.06	2.12	1.18
latent transforming growth factor beta binding protein 2	Ltbp2	2.67	3.25	6.17E-03	4.16	3.72E-16	7.5	6.18	1.75	1.22
heme oxygenase 1	Hmox1	4.59	5.33	2.45E-06	2.05	1.67E-17	3.54	—	1.86	1.25
TSC22 domain family, member 1	Tsc22d1	3.82	4.5	1.12E-03	2.1	1.82E-11	2.77	2.59	1.62	1.04
glycoprotein nmb	Gpnmb	3.21	3.83	2.24E-04	2.35	8.47E-08	2.55	—	2.22	1.26
ATPase Na+/K+ transporting subunit alpha 2	Atp1a2	−3.47	−3.38	7.10E-06	−2.23	1.25E-04	0.37	0.32	−1.66	—
glutathione S-transferase zeta 1	Gstz1	−1.97	−1.76	1.18E-03	−2.38	9.02E-23	0.34	-	−1.41	—
collagen type XVIII alpha 1 chain	Col18a1	2.59	3.17	1.02E-05	1.61	8.08E-13	-	-	3.22	—
cysteine and glycine-rich protein 2	Csrp2	3.1	3.72	1.08E-02	2.02	3.69E-05	-	-	1.81	2.09
S100 calcium-binding protein A4	S100a4	2.43	2.99	1.11E-04	1.93	1.55E-04	-	-	1.68	1.04
ankyrin repeat domain 2	Ankrd2	4.04	4.74	3.28E-06	2.49	1.27E-02	-	-	1.61	—
procollagen lysine, 2-oxoglutarate 5-dioxygenase 2	Plod2	6.89	7.82	6.73E-07	2.3	3.43E-15	-	3.36	1.91	1.19
tenascin C	Tnc	5.18	5.96	7.37E-03	2.53	3.08E-07	-	-	1.91	

## 4 Discussion

We have described the RV proteome of control and MCT rats with clear hemodynamic evidence of RVF and identified a catalogue of differentially regulated proteins in this tissue. While only a small subset of these proteins passed a stringent z-score filter, we were able to rely upon our previously published transcriptome data from an independent set of MCT rats (Tian et al., 2020b) in order to widen the net, in efforts to identify regulated transcript-protein pairs that are important in RVF. We have focused on a subset of 1,277 proteins that are significantly differentially regulated in the MCT RV compared to control. The list of proteins includes many that fail the stringent z-score filter of ± 1.96. Consequently, we compared these proteins to our previously published RNAseq data (Potus et al., 2018) and identified 410 proteins that have corresponding changes in their gene expression patterns in the RV of an independent group of animals. These 410 translated genes can be considered to have been validated, meaning they are consistently found to be regulated in RVF associated with PAH, by virtue of their detection using independent technologies in independent preclinical PAH models, conducted by independent research groups (Potus et al., 2018; Park et al., 2021). We compared these 410 protein: transcript pairs against a recently published transcriptomic paper by (Park et al., 2021) where RNAseq was performed on the RVs of male MCT rats. In Park’s study 387 (94%) of the 410 targets we identified were also differentially regulated in the MCT RV (Supplementary Figure S2).

We further filtered our data to identify only proteins satisfying the most stringent statistical test (as defined by a qval-Limma *p* < 0.05, z-score ± 1.96) that were also highly enriched at the gene expression level (as defined by a corrected *p*-value<0.05, fold-change >1.5-fold). Of these 15 highly enriched targets, we saw that 14 were concordantly regulated in the monocrotaline model, as reported in publications by independent groups (Suen et al., 2019; Park et al., 2021) and nine were similarly regulated in a different model of PAH, SuHx induced PAH in the Fisher rat resulting in severe RVF and PAH (17). Remarkably, despite differences in rodent strain, PAH model and modalities for mRNA measurement, the protein: transcript pairs that we identified were similarly regulated in these independent studies. To place these highly enriched targets into context, based on the literature, we will briefly discuss each of these robust 15 transcribed proteins with reference to the biological plausibility that they participate in either adaptive or maladaptive mechanisms of RVF in PAH. It is noteworthy that most of these proteins were upregulated with only two downregulated transcripts.

Atrial natriuretic peptide (ANP, encoded by the Nppa gene; upregulated in MCT RV: protein = 7.55-fold, transcript = 4.19-fold) is a hormone secreted by the heart in response to volume overload that mediates cardiovascular homeostasis through increased renal sodium excretion. ANP is an important clinical diagnostic marker of congestive heart failure (Brandt et al., 1993), and is known to be elevated in patients with PAH, reflecting RV decompensation (Wiedemann et al., 2001). In Japan, carperitide (a synthetic ANP) is approved as a therapeutic for acute decompensated heart failure (Nomura et al., 2008). Increased RV expression of Nppa has previously been demonstrated in the MCT rat (Radik et al., 2019) and also in the Fisher Sugen5416 + hypoxia (Suen et al., 2019).

Thrombospondin-4 (Thbs4; upregulated in MCT RV: protein = 5.56-fold, transcript = 4.4-fold) is an extracellular matrix protein that is involved in tissue remodelling through cell-matrix interactions and its gene expression is upregulated acutely in the LV in response to pressure overload (Mustonen et al., 2008). Thbs4 is both proangiogenic (Muppala et al., 2015) and proinflammatory (Frolova et al., 2012), driving macrophage accumulation and supporting macrophage differentiation (Rahman et al., 2020).

Periostin (Postn; upregulated in MCT RV: protein = 3.83-fold, transcript = 4.56-fold) is also concordantly enriched at transcript and protein levels in the MCT RV, validating previous work by our group and others (Potus et al., 2018). Periostin is involved in fibroblast proliferation and hypertrophic scar formation in skin (Crawford et al., 2015). In the lungs of both human and animal models of PAH, Postn expression is increased and correlates with PH severity (Mura et al., 2019; Nie et al., 2020). Delivery of siRNA targeting Postn intraperitoneally in the SuHx mouse model decreases RVSP and RV hypertrophy, supporting the pathophysiologic relevance of this protein in PAH-associated RVF(37). Postn expression is also increased in the LV in a rat model of postinfarction heart failure (Tulacz et al., 2013). In the MCT model, RV fibrosis is increased and in part reflects an epigenetically driven, metabolically mediated, activation of fibroblast proliferation (Tian et al., 2020b). Periostin is capable of inducing iNOS expression and NO production in RV fibroblasts in MCT rats, a process mediated by ERK1/2, JNK and NF-KB signalling in the MCT rat (Imoto et al., 2018).

Latent transforming growth factor beta binding protein 2 (Ltbp2; upregulated in MCT RV: protein = 2.67-fold, transcript = 4.16-fold) is involved in the regulation of TGF-β and is significantly elevated in the LV from heart failure patients (Bai et al., 2012). Microarrays from two groups demonstrate Ltbp2 mRNA is significantly increased in failing human LVs (Gabrielsen et al., 2007; Wei et al., 2008). Ltbp2 is also upregulated in the LV of a rat model of dilated cardiomyopathy and congestive heart failure (Pang et al., 2020). Importantly, knockdown of Ltbp2 in this model decreases reactive oxygen species and malondialdehyde, and this reduction in oxidative stress is associated with a decrease in LV fibrosis and adverse remodelling, indicating a role for Ltbp2 in the pathogenesis of dilated cardiomyopathy. Plasma Ltbp2 levels predict all-cause mortality, particularly pulmonary death, in patients with acute dyspnoea (including patients with chronic obstructive pulmonary disease and pneumonia acute pulmonary embolism) (Breidthardt et al., 2012). While the role of Ltbp2 in PAH associated RVF has not been studied, a recent study confirmed that a 2bp insert into this gene resulted in an infant with clinical features that included pulmonary hypertension with right ventricular impairment (Vollbach et al., 2021).

Heme oxygenase 1 (Hmox1; upregulated in MCT RV: protein = 4.59-fold, transcript = 2.05-fold) enzymatically degrades the pro-oxidant heme producing carbon monoxide (an anti-inflammatory, antiapoptotic vasodilator and antioxidant) (Fredenburgh et al., 2007). Although Hmox1 is downregulated in the RV of a porcine systemic-to-pulmonary shunt model of PAH (Belhaj et al., 2013), Hmox1 is upregulated in the RV and lungs in response to high dose MCT treatment in mice (600 mg/kg), and inhibition of Hmox1 using a specific inhibitor of Hmox1, SnPP IX in these animals actually leads to a promotion of inflammatory changes in the lung and increased RV hypertrophy. The authors suggest that Hmox1 induction in the lungs may supress myocardial hypertrophy in MCT induced PAH (Goto et al., 2002). This study is supported by a second that suggests in the lung, Hmox1 induction using hemin attenuates inflammation (Shimzu et al., 2008). Cardiac-specific Hmox1 overexpression model preserves left ventricular contractile performance in hearts subjected to ischemia-reperfusion injury using a Langendorff model (Yet et al., 2001). Hmox1 may therefore have a protective role in the RV, expressed in a futile effort to rescue hypertrophy. However, this cardioprotection might be context specific or temporal, since cardiac specific Hmox1 overexpression in mice attenuates isoproterenol induced cardiomyopathy but was correlated with age related, spontaneous heart failure and chronic pressure overload (Allwood et al., 2014). Context dependency is also indicated because Hmox1 is associated with ferroptosis in cardiomyopathy induced by doxyrubicin (Fang et al., 2019). More work is required to understand the role that Hmox1 plays in right heart failure.

Transforming Growth Factor Beta-1-Induced Transcript 4 Protein (Tsc22; upregulated in MCT RV: protein = 3.82-fold, transcript = 2.1-fold) Tsc22 regulates alpha smooth muscle actin, PAI-1, fibronectin and collagen I, contributing to myocardial fibrosis (Yan et al., 2011). Tsc22 is also upregulated in the left ventricle of spontaneously hypertensive rats (SHR), in experimental myocardial infarction models and in models of LVH caused by chronic pressure overload driven by either arginine vasopressin or angiotensin II (Kelloniemi et al., 2015). While adenoviral overexpression of TCS22 failed to significantly regulate many heart failure-relevant transcripts (including brain natriuretic peptide, Anp, Il6 and Col1a), it did elicit a robust increase in Col3a1 in the LV. The role of Tsc22 in RVF associated with PAH RVF is unknown.

Transmembrane glycoprotein NMB (Gpnmb; upregulated in MCT RV: protein = 3.21-fold, transcript = 2.35-fold) is a macrophage-specific, glycosylated, transmembrane protein. Gpnmb expression is enriched in inflammatory macrophages where it acts as a negative regulator of inflammation. Overexpression of Gpnmb in RAW264.7 cells reduces the production of various cytokines and mediators, including Interleukin-6 and nitric oxide, in response to LPS (Ripoll et al., 2007). Furthermore, knockdown of Gpnmb in bone marrow-derived macrophages inhibits M2 polarization (Zhou et al., 2017). There is emerging evidence for the role of macrophages in RVF in PAH (Sydykov et al., 2018) but the role of Gpnmb remains unstudied.

Collagen XVIII, alpha 1 (Col18a1; upregulated in MCT RV: protein = 2.59-fold, transcript = 1.61-fold) encodes a polypeptide that can be proteolytically processed to generate the antiangiogenic factor, endostatin. Elevated circulating levels of endostatin correlate with disease severity in PAH and higher mortality in PAH; conversely, a missense variant in Col18a1 is associated with reduced mortality (Damico et al., 2015). In infants with PH, circulating endostatin is significantly associated with RV dysfunction (Griffiths et al., 2020). In the heart, microarrays and qPCR validation have revealed a robust increase in expression of Col18a1 in the LV in a rat model of postinfarction heart failure (Tulacz et al., 2013).

Cysteine and glycine-rich protein 2 (Csrp2; upregulated in MCT RV: protein = 3.1-fold, transcript = 2.02-fold) is part of the zinc-binding LIM domain family of proteins and is highly expressed in vascular smooth muscle (Knöll et al., 2002). Although mice lacking Csrp2 survive, they exhibit cardiac hypertrophy (Sagave et al., 2008). The role of Csrp2 in RVF associated with PAH is unknown.

S100 calcium-binding protein A4 (S100a4; upregulated in MCT RV: protein = 2.43-fold, transcript = 1.93-fold) is part of a family of calcium binding proteins that serve as a metastasis promoting protein. Transgenic overexpression of S100a4 in mice results in plexiform lesions, adverse pulmonary vascular remodelling and increased right systolic pressure in female, but not male mice (Dempsie et al., 2011). In the MCT model however, the expression of S100a4 is significantly upregulated in the pulmonary artery, lung and the RV of male rats (Song et al., 2018), which our data confirm at both the transcript and protein level.

Ankyrin repeat domain 2 (Ankrd2; upregulated in MCT RV: protein = 4.04-fold, transcript = 2.49-fold) is a member of the muscle ankyrin repeat protein (MARP) family of proteins and is expressed primarily within skeletal muscle, where it is upregulated in response to mechanical stretch (Kemp et al., 2000) and its transcription is induced by reactive oxygen species (Cenni et al., 2019). Ankrd2 is dispensable for cardiac function at baseline and following pressure overload (Bang et al., 2014), and its function in the decompensated MCT RV remains unclear.

Procollagen-Lysine,2-Oxoglutarate 5-Dioxygenase 1 (Plod1; upregulated in MCT RV: protein = 6.89-fold, transcript = 2.3-fold) encodes the enzyme lysyl hydroxylase which catalyses the conversion of lysine to hydroxylysine. Collagen biosynthesis is dependent upon the appropriate hydroxylation of lysyl residues. Hydroxylysine acts as an attachment site for carbohydrates and helps stabilize collagen crosslinks in order to enhance tensile strength of collagen fibrils (Rautavuoma et al., 2004). Increased Plod1 in the failing MCT RVs may promote excessive fibrosis, a hallmark of the MCT model (Potus et al., 2018).

Tenascin-C (Tnc; upregulated in MCT RV: protein = 5.18-fold, transcript = 2.53-fold) is an extracellular matrix molecule involved in tissue remodelling. Primarily expressed during embryonic development, tenascin-C is re-expressed during inflammatory pathologies (Imanaka-Yoshida, 2012). Tenascin-C is upregulated and pathologically relevant in the pulmonary vasculature in PAH (Jones and Rabinovitch, 1996; Cowan et al., 2000). In the rat, mRNA expression of Tnc is also significantly upregulated in the RV of MCT treated animals (80 mg/kg) compared to both control animals and rats who received a lower MCT dose (30 mg/kg). Tenascin-C was undetectable in the control, or the low dose MCT group, but was located within the RV extracellular space in the high-dose MCT group. In the same study, plasma tenascin-C was only detectable in high-dose MCT, indicating that Tnc is a candidate mediator of RVF (Hessel et al., 2009).

While most highly regulated concordant protein: transcript pairs were upregulated in RVF several were downregulated; for example, ATPase Na+/K + Transporting Subunit Alpha 2 (Atp1a2; downregulated in MCT RV: protein = 3.47-fold, transcript = 2.23-fold). Atp1a2 is a component of the ATPase enzyme that catalyzes the hydrolysis of ATP. ATP1a2 is considered a minor isoform expressed in cardiac myocytes. However, the a2 subunit may be maladaptive because Atp1a2 knockout mice are protected from pressure overload-mediated cardiac dysfunction (Rindler et al., 2013). The effects of the observed downregulation of Atp1a2 in PAH RVF are unknown.

Glutathione S-Transferase Zeta 1 (Gstz1; downregulated in MCT RV: protein = 1.97-fold, transcript = 2.38-fold) is an enzyme located in both the cytosol and mitochondria which catalyzes the final steps of phenylalanine and tyrosine synthesis. Gstz1 also controls the biotransformation of dichloroacetate (DCA) to glyoxylic acid in the mitochondrion (Li et al., 2011). This is directly relevant as DCA has therapeutic efficacy for RVF in PAH both in patients (Michelakis et al., 2017) and rodents (Michelakis et al., 2002; Piao et al., 2010b; Piao et al., 2013). The role of Gstz1 in RVF is unknown.

We also identified several potentially relevant proteins that miss z-the score cut-off for protein expression, but which were significantly regulated at the transcript level. For example, Col1a1 is upregulated in the MCT RV, consistent with increased fibrosis in the right ventricle and heart failure. A recent meta-transcriptome paper demonstrated that this transcript is a robust biomarker for human heart failure progression in the left ventricle (Hua et al., 2020). Although Collagen alpha III is known to be regulated at both the mRNA (Potus et al., 2018) and the protein (Al-Qazazi et al., 2022) level in the male RV of MCT induced rats, we did not observe Col3a1 protein changes in our proteomic data. We do not have data to determine whether this reflects an experimental issue (such as inadequate sample size) or a statistical false discovery issue. Follistatin-like protein 1 (Fstl1) is significantly upregulated in the MCT RV but also misses the z-score cut-off for robust, statistical significance. Fstl1 is a secreted glycoprotein that is reportedly cardioprotective (Tanaka et al., 2016). Deletion of this gene results in excessive smooth muscle actin associated with atrial endocardia, heart valves, veins and micro-vessels through SMAD3 activation, but also collagen deposits in the atria (Jiang et al., 2020). Serum levels of Fstl1 are elevated in human patients with pulmonary hypertension related to COPD, and in the lungs of hypoxic mice with high right ventricular systolic pressure (RVSP) and RVH (Zhang et al., 2017). In the same study, haploinsufficiency of Fstl1 in hypoxic mice raised RVSP and RVH was rescued with Fstl1 overexpression. The fact that this protein and its transcript are upregulated in the RV in our data set suggests ongoing RV compensatory mechanisms may be in play.

Our analysis revealed that the high-level functions of the proteome closely mirrored the Gene Ontology analysis from our previously published transcriptome data (Potus et al., 2018). Mitochondrial metabolism, cellular respiration and purine metabolism are enriched functions within the proteome in the failing RV in rats with MCT induced PAH. When we filtered down to those 410 proteins whose encoding genes are also differentially regulated, the same functional groups were evident. Importantly, 22 members of the NADH-ubiquinone oxidoreductase family are downregulated in the failing RV in response to MCT. Also, seven genes encoding subunits of ATP synthase are downregulated at both transcript and protein levels within our data.

Interestingly, the transcript encoding the protein ATPase family gene 3-like 2 (Afg3l2) is downregulated. Afg3l2 is located in the inner mitochondrial membrane. Afg3l2 is an m-AAA mitochondrial protease and degrades misfolded proteins, regulates ribosome assembly and also regulates the processing of the fusion mediator OPA-1. Mutations of this gene are known to cause neurodegenerative diseases, including spinocerebellar ataxia (Di Bella et al., 2010) and optic atrophy (Charif et al., 2015). The role of m-AAA proteases is to ensure appropriate mitochondrial protein homeostasis which in turn ensures normal mitochondrial fusion and efficient oxidative phosphorylation. Inactivation of Afg3l2 in mice results in reduced activity of complex I, III, and IV (Pareek and Pallanck, 2020) and mitochondrial fragmentation in Purkinje cells (Almajan et al., 2012). One example of the function of this m-AAA protease is its ability to degrade non-assembled protein subunits of the mitochondrial calcium uniporter (MCU), specifically, essential mitochondrial uniporter regulator (EMRE). EMRE is part of the MCU pore and allows the transport of Ca2+ across the inner membrane of the mitochondria. Excess EMRE can result in a Ca2+ overload and cell death (König et al., 2016) and so the loss of m-AAA is the same as overexpression of EMRE. We have recently demonstrated the importance of the MCU in pulmonary artery smooth muscle cells and demonstrate that decreased expression of MCU resulted in a PAH phenotype including mitochondrial fragmentation and Warburg-like metabolism. In contrast, increasing MCU expression rescued these cells, decreasing proliferation, and inhibiting mitochondrial fission (König et al., 2016). There may also be tissue/cell specific actions here, since in both early- and end-stage pressure overload induction of heart failure, MCU is actually upregulated and MCU inhibition confers cardioprotection (Yu et al., 2018). While EMRE is a critical protein for normal function of the MCU complex, EMRE overexpression results in non-productive subcomplexes (Tsai et al., 2017), a problem avoided by increased EMRE degradation. Finally, loss of Afgl2 expression interferes with processing of PA-1, reducing expression of the long isoform of OPA1 (L-OPA1), which inhibits mitochondrial fusion and triggers mitochondrial fragmentation. While the precise role of Afg3l2 in MCT induced RVF is currently unknown, the observed downregulation would be expected to impair MCU complex function and promote mitochondrial fission. These effects might lead to apoptosis and generation of ROS. Our multiomic data suggest that the failing RV has a profound dysregulation of proteins that control mitochondrial function and cellular respiration. This is consistent with prior demonstrations that the PAH RV has a profoundly deranged mitochondrial phenotype (Lee and Jung, 2018; Potus et al., 2018; Tian et al., 2018; Tian et al., 2020b).

Our data also demonstrate that many regulated proteins are involved in purine metabolic processes. Consistent with this hypothesis, our previous metabolomics study identified altered purine metabolism in the MCT RV (Prisco et al., 2020). Purines are heterocyclic aromatic organic compounds that make up the two groups of nucleotide bases (adenine and guanine), and are components of adenosine triphosphate (ATP), guanosine triphosphate (GTP), cyclic adenosine monophosphate (cAMP) and nicotinamide adenine dinucleotide (NADH). Purines are ultimately catabolized by cells into uric acid, which is a powerful antioxidant and a scavenger for reactive oxygen species (Ames et al., 1981). High levels of uric acid are a marker for impaired oxidative metabolism (Nagaya et al., 1999). In patients with idiopathic PAH, serum uric acid levels are not only higher than controls, but there is a correlation between uric acid and mean PA pressure, as well as an inverse correlation with RV function (Zhang et al., 2013).

In order to place our findings into appropriate context, some limitations require emphasis. Firstly, we acknowledge that our reuse of surgical sham animals from a prior experiment may increase false positive discovery of targets, beyond rates normally associated with omic data. Relative to untreated control rats used in our transcriptomics experiment (Tian et al., 2020b), the surgical sham animals used in the current study were subject to the anesthetic, sham surgery, and a recovery period. However, because our experimental approach compared a new cohort of monocrotaline rats not only with these sham rats but also with studies reported by multiple independent groups, we are satisfied that the identified regulated proteins are valid. To further confirm the validity of our control group, we selected three regulated transcripts (Postn, Nppa and Ltbp2) and demonstrated that there were no significant differences between conventional animals and animals subject to sham surgery. Our reuse of an existing control group of rats is consistent with the highest standards of animal welfare and fits within the 3R framework of animal experimentation: replacement, reduction and refinement (i.e. avoidance of unnecessary use of animals in experiments when existing data can be repurposed). Secondly, while we were not in control of the animal preparation, hemodynamics or molecular biology in the studies from other laboratories that we mined for comparative transcriptome data, we trust that these peer reviewed datasets were well controlled. An advantage of our approach (which corroborates our multiomic findings with reported findings in the literature) is that the likelihood of false discovery (driven by different animal models, and different data types) is mitigated by the focus on those regulated protein: transcript pairs that are similarly regulated across studies. Third, our multiomic data are derived from intact RVs and did not enrich for specific cell-types within the myocardium. For this reason, we cannot comment on whether the regulated proteins come from a particular cell type (fibroblast, cardiomyocytes, neurons, and vascular cells). Future work should focus on cell-type specific transcriptomics and proteomics in PAH RV. We also want to draw attention to the percentage overlap between the total significant proteins, and our previously published RNA data. While the 32% overlap of 410 protein:transcript pairs is low, we suggest that a rigorous approach to analysis, combined with the multiple test correction issues in both datasets have resulted in a high degree of false negatives. We therefore encourage readers interested in putative RV targets to explore our raw data and look for protein:transcript pairs that satisfy lower statistical thresholds. We also acknowledge that this study was performed exclusively on male animals. A sex paradox exists in PAH which is not well explored. While females are up to 4-fold more likely to develop PAH than male patients, they also have better survival rates (Humbert et al., 2006; Humbert et al., 2010; Escribano-Subias et al., 2012; Lee et al., 2012; Mair et al., 2014). Here, we have only investigated male rat RVs because we believe they may have a more extreme RV phenotype, since it is probable that the sex based discrepancy in survival in human PAH patients is explained by a more maladaptive response by the RV to pressure overload in males (Jacobs et al., 2014). Future omic experiments in the female RV will allow us, and others to compare the RV transcriptome and proteome between the sexes, so that we can further unpick this paradox. In this study, we also did not acquire LV function using echocardiography; however, our PV-loop catheterization data show a significant reduction in left ventricular systolic pressure (LVSP) and a non-significant decrease in left ventricular end-diastolic pressure (LVEDP) in MCT rats when compared to controls (These data are now added to Supplementary Figure S3). This data is in line with our group’s previous work, in which Tian and Xiong et al., 2020 reported similar hemodynamic data (Tian et al., 2020a). Finally, Pinto et al., 2007 show that in MCT-induced PAH, baseline LV dysfunction was present in MCT rats only at 6 weeks post-treatment, which is a sign that can also be observed in patients with chronic PAH due to the compression and atrophy of the LV that results from continuous RV afterload (Correia-Pinto et al., 2009). These data in aggregate suggest that the MCT drug effect primarily impacts the RV rather than LV. Indeed, we have previously observed that the Nppa is expressed exclusively in the decompensated RV, but not the LV (Al-Qazazi et al., 2022). We have reviewed the literature in an effort to identify compartment specific gene/protein expression, and reanalyzed the one available dataset where PAH was induced with MCT, and found that of the targets that map between platforms (microarray and RNAseq) (Kittleson et al., 2005), none of our most robustly regulated RV genes were significantly regulated in that study of the LV. We also note caveats to the utility of that prior study, namely that PAH was induced in Wistar rats, relied on microarrays with a low sample size (n = 2). Finally, we did not manipulate the function of the 15 most regulated protein: transcript pairs in the RV. This research is important and feasible but is beyond the scope of our initial goal, an unbiased definition of highly regulated and conserved changes in the RV multi-ome. We also identified 867 proteins that are differentially expressed in the absence of any change in transcriptional activity and 2,133 regulated transcripts for which no significant change in expression of the corresponding protein was detected. Our analysis of the MCT RV transcriptome and proteome also revealed 18 targets that were contra-regulated, e.g. regulated in different directions at the protein versus transcript level (Supplementary Figure S4). The function of this protein is to facilitate promotor-dependent transcription of the mitochondrial genome by the mitochondrial polymerase, POLRMT. Tfam has been shown to have a stimulatory effect on transcription and is a critical player in mitochondrial transcription (Posse et al., 2015). The lack of concordance between these transcripts and proteins may reflect delays between transcription and translation as well as proteasomal regulation of protein expression and epigenetic regulation of mRNA expression.

In conclusion, we performed a layered transcriptomic, proteomic analysis of the MCT RV in rats with PAH-associated RVF. We demonstrated the robust regulation of 1,277 proteins in PAH associated with RVF, 410 of which also have concordantly regulated transcripts. We have validated these gene/protein pairs using the literature and demonstrated that many of these targets are implicated either in other models of PAH, or in other forms of heart failure. Taken together, the molecular phenotype of the PAH RV has an overarching contribution from dysregulation of mitochondrial metabolism. Many of the dysregulated pathways also favour excessive RV fibrosis. Integration of transcriptome and proteome expression profiles within the RV of this decompensated model of PAH provides the most comprehensive picture to date of the molecular events occurring within the decompensated RV in experimental PAH.

## Data Availability

The datasets presented in this study can be found in online repositories. The datasets analyzed for this study can be found in the Gene Expression Omnibus GSE119754.
