# Supplementary figures and images for: An integrated proteomic and transcriptomic signature of the failing right ventricle in monocrotaline induced pulmonary arterial hypertension in male rats
Source: Front Physiol. 2022 Nov 1;13:966454. doi: 10.3389/fphys.2022.966454 (PMC9664166; doi:10.3389/fphys.2022.966454)

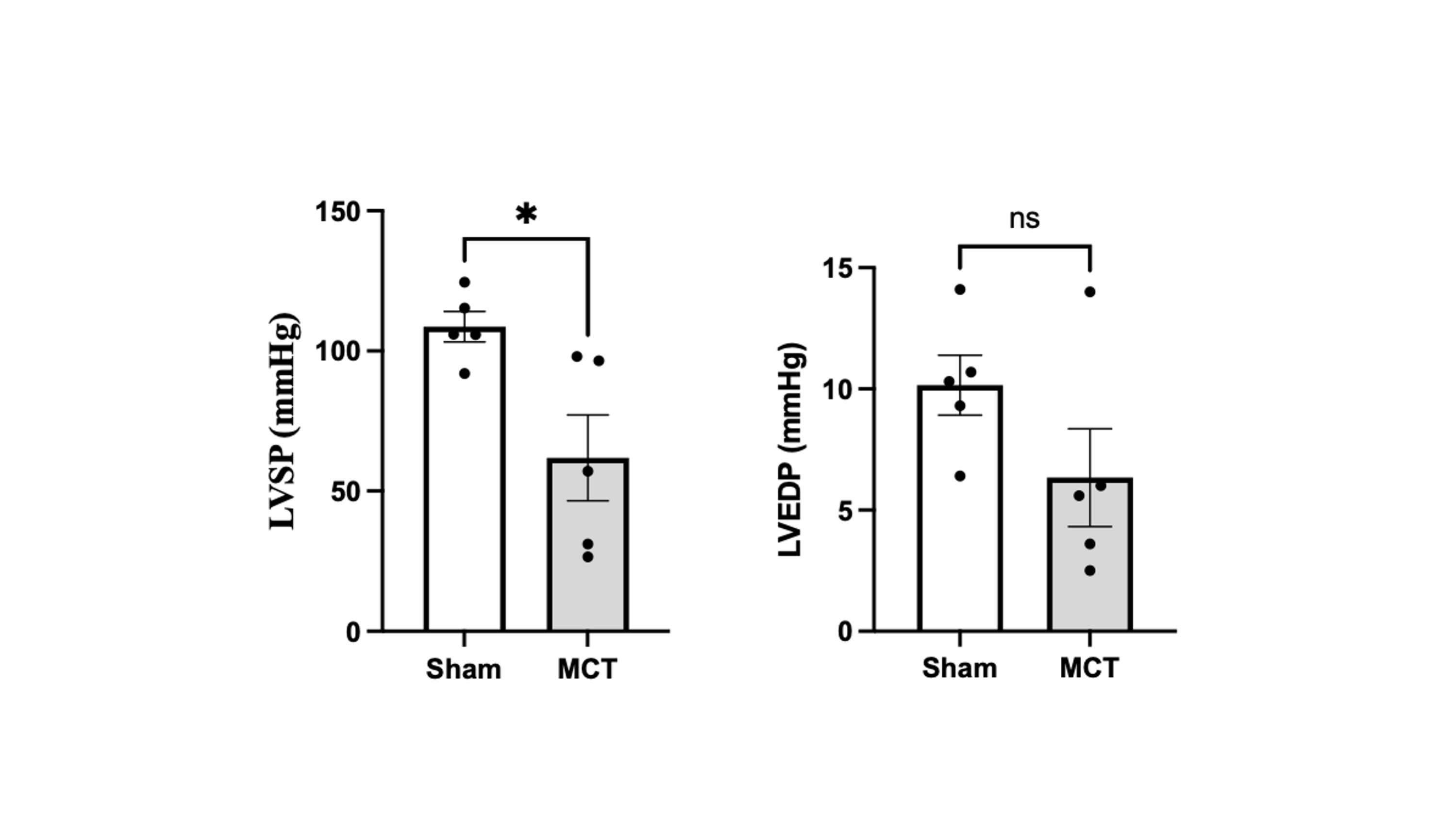

Supplement: Supplementary file 1 [file Image3.tiff]

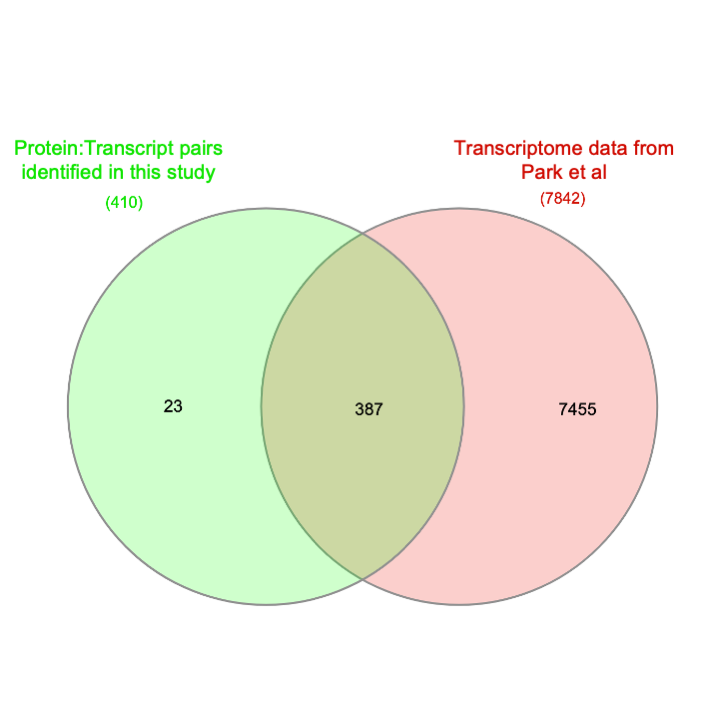

Supplement: Supplementary file 2 [file Image2.tif]

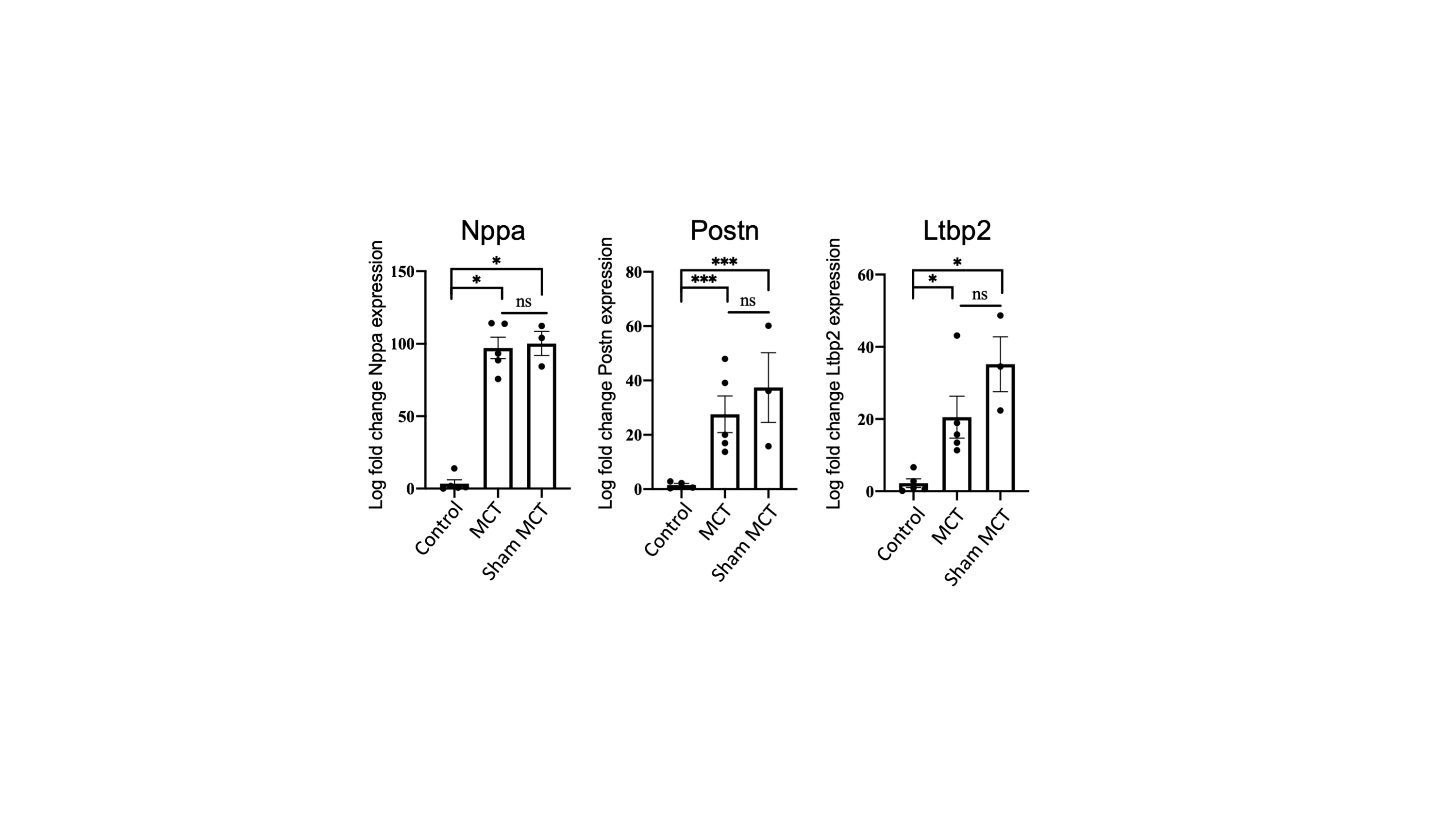

Supplement: Supplementary file 3 [file Image1.tif]

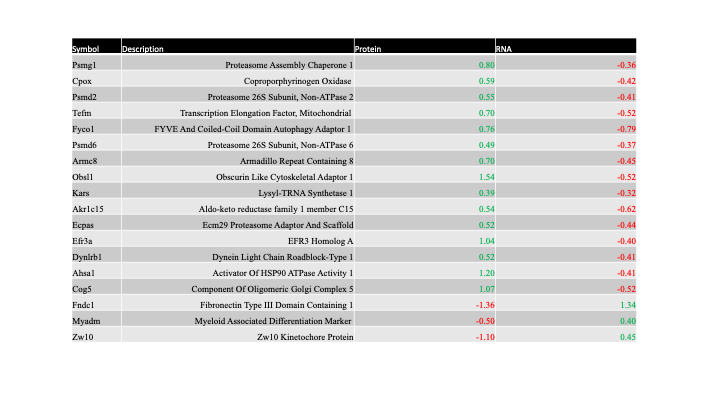

Supplement: Supplementary file 5 [file Image4.tiff]
